# Association between physical performance and incidence of end-stage renal disease in older adults: a national wide cohort study

**DOI:** 10.1186/s12882-021-02291-4

**Published:** 2021-03-10

**Authors:** Hee-won Jung, In Young Choi, Dong Wook Shin, Kyungdo Han, Jung Eun Yoo, Sohyun Chun, Yongjin Yi

**Affiliations:** 1 Division of Geriatrics, Department of Internal Medicine, Asan Medical Center, University of Ulsan College of Medicine, Seoul, South Korea; 2 Total Healthcare Center, Kangbuk Samsung Hospital, B1, Samsung Main B/D, 67, Sejong-daero, Jung-gu, Seoul, 04514 South Korea; 3 Department of Digital Health, SAIHST, Sungkyunkwan University, 81 Irwon-Ro, Gangnam-gu, Seoul, 06351 South Korea; 4 Department of Statistics and Actuarial Science, Soongsil University, Seoul, South Korea; 5 Department of Family Medicine, Healthcare system Gangnam Center, Seoul National University Hospital, Seoul, South Korea; 6 International Healthcare Center, Samsung Medical Center, Seoul, South Korea; 7 Division of Nephrology, Dankook University Hospital, Cheonan-si, Chungcheongnam-do South Korea

**Keywords:** Frailty, Physical function, Chronic kidney disease, Morbidity

## Abstract

**Background:**

Physical frailty has previously been associated with adverse clinical outcomes in patients with end-stage renal disease (ESRD). This study aimed to determine whether impaired physical performance at baseline is associated with the incidence of ESRD, using a nationwide database.

**Methods:**

The timed up-and-go (TUG) test was used to assess physical frailty in 1,552,781 66-year-old individuals, using health examination database records from the Korean National Health Insurance Service. As a primary endpoint, incident ESRD was defined operationally using healthcare claims data from the Korean Health Insurance Review and Assessment Service.

**Results:**

Our results showed that baseline kidney function was significantly worse in individuals with TUG results of > 10 s compared to individuals with an intact TUG performance (≤10 s). Kaplan-Meier analysis showed a stepwise dose-response relationship between baseline physical performance and the incidence rate of ESRD (log-rank test *P*-value of < 0.001). An increasing ESRD incidence rate trend with poor physical performance remained significant after adjusting for characteristics such as baseline glomerular filtration rate and proteinuria.

**Conclusion:**

Poor baseline physical performance was associated with an increased risk of ESRD, suggesting possible interactions between systemic frailty and vascular aging processes.

## Background

Due to global population aging and a rising number of patients with chronic multimorbidities such as diabetes mellitus (DM) and hypertension (HT), effective healthcare for patients with chronic kidney disease (CKD) has become more urgent. Globally, end-stage renal disease (ESRD), a state of advanced CKD requires either kidney transplantation or renal replacement therapy, has been shown to affect patient quality of life and lead to morbidity and mortality [1, 2]. A United States study estimated the cumulated lifetime risk of developing ESRD to be approximately 2% among individuals of European ethnicity and 7% among those of African-American ethnicity, when calculated at 20 years of age [3]. A recent study suggested that the lifetime incidence of ESRD at birth was 3.6% in the United States [4]. Furthermore, treatment costs have been estimated to be considerably higher for patients with ESRD than for patients with common malignancies [3].

Worldwide, the causes of CKD vary considerably; however, chronic diseases such as DM and HT have been reported to be main causes of CKD in high- and middle-income countries [2]. These chronic diseases and metabolic risk factors, such as cigarette smoking and obesity, have been reported to be factors promoting glomerulosclerosis, which eventually leads to decreased kidney function [5]. Accelerated features of the aging phenotype, including vascular calcification, cachexia, and frailty, have been observed in relation to decreasing kidney function, suggesting cross-links between kidney function and systemic aging, especially given the vascular nature of the kidney [6, 7].

Frailty is a common geriatric syndrome and a marker of biological age that is defined as a state of decreased physiological reserve and increased vulnerabilities for possible stressors with aging, and is a well-known risk factor for morbidity, mortality, and functional decline in older adults [8, 9]. Several studies have shown that frailty is associated with systemic inflammation and phenotypic vascular aging that commonly occurs with chronic diseases [10–13]. Furthermore, recent studies have suggested that reducing systemic inflammation associated with aging, both in an accelerated aging mice model and in naturally aged mice, can lead to an improvement in frailty in rodents [14, 15]. These results suggest that frailty is a biological construct that may reflect the systemic aging status of organisms.

In patients with ESRD, one study reported that frailty was common in approximately 50% of study patients aged ≥65 years old [16], and an even younger population with ESRD showed physical frailty [17]. A study from Korea reported that baseline frailty in patients with ESRD undergoing hemodialysis adversely affected composite outcomes including all-cause mortality and hospitalization [18]. Similarly, a study from the United States showed that frailty was associated with adverse outcomes in patients having undergone kidney transplantation [17, 19]. To our knowledge, despite these outcomes and the significance of frailty, the role of frailty in the development of ESRD in older adults has not been reported to date. This study aimed to determine whether physical frailty measured using the timed ‘up-and-go’ (TUG) test at baseline was associated with the incidence of ESRD in Korean older adults aged 66 years who had participated in the National Screening Program for Transitional Ages (NSPTA).

## Methods

### Study setting and population

In this study, we used data derived from the Korean National Health Insurance Service (KNHIS) database. The KNHIS is a nationwide universal healthcare insurer that serves virtually the entire Korean population, comprising 96.9% of those paying into the insurance system and the underserved population that receives medical aid. This data is publicly available from https://nhiss.nhis.or.kr/bd/ay/bdaya001iv.do.

The KNHIS database contains information on demographic factors, such as age, sex, death date, and the results of the national health screening program, as well as information on the utilization of medical facilities, International Classification of Diseases 10th Revision (ICD-10) codes, and reimbursement data for procedures and medicines prescribed in both outpatient and inpatient settings.

The NSPTA is a special part of the national health screening program for 66-year-old individuals that includes a geriatric assessment [20] in the National Health Screening Program (NHSP), which is provided by the KNHIS. The NSPTA consists of a health examination that is offered during a routine NHSP and includes a lifestyle assessment questionnaire, anthropometric measurements, and laboratory tests, as well as geriatric functional examinations for physical performance and cognitive function.

In this study, we analyzed NSPTA data from 2009 to 2015 involving a population of 1,852,792 individuals. We excluded 238,865 individuals registered with the National Disability Registration System having a baseline disability (e.g. visual/hearing disability, mental/cognitive disability, and amputation or spinal cord injury) as they were unable to perform the TUG test. We excluded individuals with ESRD at baseline (*n* = 1363) or with a baseline estimated glomerular filtration rate (eGFR) of < 15 mL/min/1.73 m^2^ (*n* = 7544). Finally, we excluded individuals with missing TUG test records (*n* = 1363) or other covariables (e.g. smoking status, *n* = 26.304), and the final study population comprised 1,552,781 individuals. This study was approved by the Institutional Review Board (IRB) of Samsung Medical Center (IRB number: SMC-2019-07-046). Informed consent requirement was waived by the Institutional Review Board because the data does not contain personal information. All methods were performed in accordance with the relevant guidelines and regulations. 

### Assessments of physical frailty

We used the TUG test to assess physical frailty. This test has previously been used as a basic functional assessment for vulnerability in older adults [22, 23] and correlates well with other frailty measures, including the 5-item Cardiovascular Health Study frailty scale score and Short Physical Performance Battery total score in the Korean population [24]. During the NSPTA, the TUG test was administered as part of the geriatric assessment in community-based clinics or hospitals using a standardized manual. Individuals were timed while they rose from a chair, walked at a comfortable pace to a line on the floor 3 m away, turned and walked back to the chair, and sat down again. Individuals were categorized into 4 groups according to their TUG test results, as follows: Group 1 (< 10 s), Group 2 (10–14.9 s), Group 3 (15–19.9 s), and Group 4 (≥20 s).

### Measurements and definitions

Cigarette smoking status was determined using a questionnaire and the responses were categorized as follows: non-smoker (never smoked), a past history of smoking, and active smoking. Alcohol consumption was categorized as follows: none (0 g/day), mild to moderate (< 30 g/day), and heavy (≥30 g/day). Regular exercise was defined as engaging in physical activity of moderate to vigorous intensity for ≥3 days per week. Information concerning comorbidities and a history of falls was obtained from responses to the questionnaire. Information concerning income levels was derived from monthly insurance premiums, as insurance contributions are determined according to income levels and not according to health risk in Korea. Blood samples for the measurement of serum creatinine (Cr), glucose, high-density lipoprotein (HDL)-cholesterol, low-density lipoprotein (LDL)-cholesterol, total cholesterol, and triglyceride levels were drawn after an overnight fast and assessed at laboratories that met national standards.

In this study, eGFR was calculated using the abbreviated Modification of Diet in Renal Disease (MDRD) formula: 175 × serum Cr (mg/dL)^–1.154^ × Age (y)^–0.203^ × (0.742 if female) [25]. For sensitivity analysis, we also used the Chronic Kidney Disease Epidemiology Collaboration (CKD-EPI) equation based on serum Cr to calculate the eGFR [26]. The level of proteinuria was assessed using the dipstick method on midstream samples and was reported using the following grades: absent, trace (±), 1+, 2+, 3+, and 4+ (corresponding to protein levels, as follows: undetectable, 10 mg/dL, 30 mg/dL, 100 mg/dL, 300 mg/dL, and 1000 mg/dL, respectively), and meaningful proteinuria was defined as dipstick results of ≥1 + .

HT was defined as the presence of at least one claim within a 12-month period under ICD-10 codes I10–11, and a history of a prescribed antihypertensive agent, or a systolic blood pressure (BP) of ≥140 mmHg or diastolic BP of ≥90 mmHg at the time of examination. DM was defined as having at least ≥1 record of International Statistical Classification of Diseases and Related Health Problems ICD codes E10–14 within a 12-month period, a history of prescription antidiabetic medication, or a fasting glucose level of ≥126 mg/dL. The presence of dyslipidemia was defined as having at least one claim within a 12-month period under ICD-10 code E78, a history of having been prescribed a lipid-lowering agent, or a total cholesterol level ≥ 240 mg/dL at the time of examination.

### Outcome definition

The primary endpoint of the study was incident ESRD, which was defined using combinations of the ICD 10th revision codes, namely, N18–19, Z49, Z94.0, and Z99.2, as recorded in the claims database, the presence of codes related to renal replacement therapies (KNHIS procedure codes O7011–O7020 or V001 for hemodialysis, O7071–O7075 or V003 for peritoneal dialysis), and/or receiving kidney transplantation (R3280) as recorded in the Korean Health Insurance Review and Assessment Service database.

### Statistical analysis

The baseline characteristics of the population groups were compared according to the TUG test results using independent t-tests for continuous variables and Chi-square tests for categorical variables. During the follow-up period starting from 2009, individuals were censored on the day of ESRD incidence, or on December 31, 2016, whichever occurred first. Incidence rates were expressed as cases per 1000 person-years. Kaplan-Meier curves were used to visualize group differences in ESRD incidence.

Hazard ratios (HRs) for the incidence of ESRD according to the TUG test groups were estimated using Cox proportional hazards regression analysis. Analysis was initially unadjusted (Model 1). We then included additional covariables, such as sex, lifestyle variables (cigarette smoking, alcohol consumption, physical activity levels, and income), in Model 2. We further adjusted for BMI and comorbidities including DM, HT, dyslipidemia, and baseline eGFR in Model 3. In Model 4, we further included the semi-quantitative status of proteinuria as a covariable in addition to variables listed in Model 3.

As individuals with low eGFR may have higher mortality than individuals with higher eGFR, and death may compete with ESRD incidence, we also performed sensitivity analysis by considering death as a competing event. The semiparametric proportional hazards model by Fine and Gray was implemented for this competing risk regression analysis [27].

Stratified analysis was performed according to baseline eGFR using both MDRD equations in 4 groups (< 30 mL/min/1.73 m^2^, 30–60 mL/min/1.73 m^2^, 60–90 mL/min/1.73 m^2^, and ≥ 90- mL/min/1.73 m^2^). Sensitivity analysis was performed using different eGFR calculation methods, such as the CKD-EPI equation.

We considered two-tailed *P*-values of < 0.05 statistically significant. All statistical analyses were performed using SAS software, version 9.4 (SAS Institute, Cary, NC, USA).

## Results

### Baseline characteristics

Of 1,552,781 older adults aged 66 years, 694,239 (44.7%) were men; 1,159,126 (74.6%) had a TUG test result of < 10 s, and 393,655 (25.4%) had a TUG test result of ≥10 s (Table 1). The presence of comorbidities, such as DM, HT, dyslipidemia, history of stroke, and myocardial infarction, was associated with a higher TUG test result. Baseline CKD status and eGFR categories using the MDRD formula with serum Cr showed a statistically significant difference between the groups dichotomized according to the TUG test results. Metabolic parameters, such as BMI, weight circumference, BP, and total cholesterol, were significantly higher in those with a higher TUG test result.

**Table 1:** Baseline characteristics of study population

n	Groups according to the TUG test results				*P*-value
	< 10 s	10–14.9 s	15–19.9 s	≥20 s	
	1,159,126		393,655		
Sex, male	536,331 (46.3)	138,662 (40.8)	16,636 (36.4)	2610 (33.9)	<.0001
Cigarette smoking					<.0001
Non-smoker	801,370 (69.1)	247,511 (72.7)	34,139 (74.7)	5842 (75.9)	
Past history of smoking	216,258 (18.7)	52,169 (15.3)	6249 (13.7)	1018 (13.2)	
Active smoker	141,498 (12.2)	40,596 (11.9)	5296 (11.6)	835 (10.9)	
Alcohol consumption					<.0001
None	820,995 (70.8)	250,469 (73.6)	34,726 (76.0)	6046 (78.6)	
Mild	283,425 (24.5)	74,619 (21.9)	9127 (20.0)	1365 (17.7)	
Heavy	54,706 (4.7)	15,188 (4.5)	1831 (4.0)	284 (3.7)	
Regular exercise, Yes	301,620 (26.0)	80,237 (23.6)	10,031 (22.0)	1516 (19.7)	<.0001
Income					<.0001
Q1 (lowest)	321,817 (27.8)	97,320 (28.6)	13,676 (29.9)	2452 (31.9)	
Q2	225,044 (19.4)	66,378 (19.5)	8921 (19.5)	1543 (20.1)	
Q3	304,506 (26.3)	90,793 (26.7)	12,050 (26.4)	1931 (25.1)	
Q4 (highest)	307,759 (26.6)	85,785 (25.2)	11,037 (24.2)	1769 (23.0)	
Comorbidity					
Dyslipidemia	444,344 (38.3)	135,290 (39.8)	18,430 (40.3)	3169 (41.2)	<.0001
Diabetes mellitus	223,381 (19.3)	70,836 (20.8)	10,206 (22.3)	1891 (24.6)	<.0001
Hypertension	599,132 (51.7)	185,989 (54.7)	26,200 (57.4)	4513 (58.7)	<.0001
History of MI	9391 (0.8)	2885 (0.9)	410 (0.9)	116 (1.5)	<.0001
History of stroke	41,371 (3.6)	14,271 (4.2)	2291 (5.0)	596 (7.8)	<.0001
CKD	115,893 (10.0)	37,670 (11.1)	5622 (12.3)	1086 (14.1)	<.0001
eGFR (using MDRD, mL/min/1.73m^2^)	84.7 ± 36.1	86.0 ± 36.2	85.0 ± 31.2	84.7 ± 41.3	<.0001
15–29	1713 (0.2)	627 (0.2)	112 (0.3)	33 (0.4)	<.0001
30–59	114,180 (9.9)	37,043 (10.9)	5510 (12.1)	1053 (13.7)	
60–89	798,406 (68.9)	219,894 (64.6)	29,361 (64.3)	4851 (63.0)	
≥90	244,827 (21.1)	82,712 (24.3)	10,701 (23.4)	1758 (22.9)	
Proteinuria					<.0001
Absent	1098,130 (94.7)	320,451 (94.2)	42,752 (93.6)	7141 (92.8)	
± (trace)	27,503 (2.4)	9018 (2.7)	1267 (2.8)	246 (3.2)	
1+	21,370 (1.8)	6882 (2.0)	1032 (2.3)	183 (2.4)	
2+	9054 (0.8)	2821 (0.8)	430 (0.9)	83 (1.1)	
3+	2543 (0.2)	913 (0.3)	162 (0.4)	37 (0.5)	
4+	526 (0.1)	191 (0.1)	41 (0.1)	5 (0.1)	
BMI (kg/m2)	24.2 ± 2.9	24.4 ± 3.1	24.6 ± 3.3	24.6 ± 3.5	<.0001
Waist circumference (cm)	82.8 ± 8.2	83.1 ± 8.3	83.6 ± 8.5	83.8 ± 8.9	<.0001
SBP (mmHg)	127.7 ± 15.0	128.4 ± 15.2	129.1 ± 15.6	128.8 ± 16.0	<.0001
DBP (mmHg)	77.4 ± 9.6	77.7 ± 9.7	78.1 ± 9.9	78.3 ± 10.1	<.0001
Serum glucose (mg/dL)	103.8 ± 25.0	104.0 ± 26.5	104.4 ± 27.6	105.8 ± 29.8	<.0001
HDL cholesterol (mg/dL)	53.8 ± 15.4	54.0 ± 16.4	54.3 ± 19.6	53.2 ± 14.2	<.0001
LDL cholesterol (mg/dL)	116.3 ± 38.7	116.2 ± 39.5	116.0 ± 38.8	116.8 ± 40.5	0.4956
Total cholesterol (mg/dL)	196.1 ± 38.4	197.0 ± 39.1	197.4 ± 40.0	197.7 ± 41.2	<.0001
TUG (sec)	7.2 ± 1.5	10.6 ± 1.1	16.0 ± 1.3	25.6 ± 12.2	<.0001
Follow-up duration (year)	5.0 ± 2.0	5.3 ± 2.1	5.6 ± 2.1	5.5 ± 2.1	<.0001

Data shown as number (percent) or mean ± (standard deviation)

*BMI* body mass index, *CKD* chronic kidney disease, *DBP* diastolic blood pressure, *eGFR* estimated glomerular filtration rate, *HDL* high-density lipoprotein, *LDL* low-density lipoprotein, *MI* myocardial infarction, *MDRD* Modification of Diet in Renal Disease, *SBP* systolic blood pressure, *TUG* timed up-and-go test

### Incidence of ESRD according to TUG performance

**Fig. 1:**
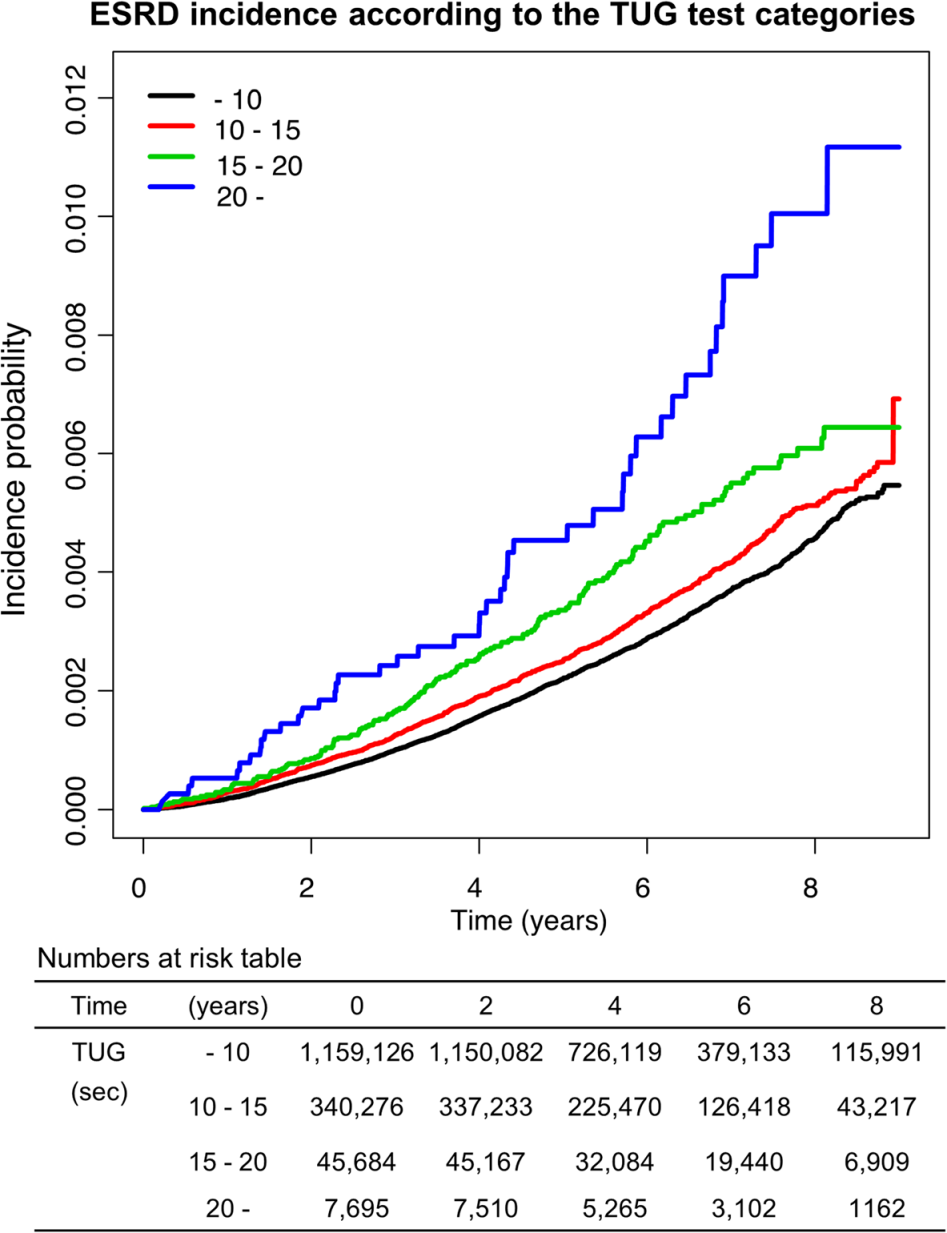
Kaplan-Meier plot and numbers at risk table for ESRD incidence according to TUG categories. ESRD, end-stage renal disease; TUG, timed up-and-go test

Figure 1 shows the cumulative incidence probability of ESRD during follow-up periods according to 4 TUG test performance categories. Using Kaplan-Meier curves, dose-response relationships between the incidence of ESRD and TUG performance categories were observed (log-rank test, *P* < 0.001).

In multivariable analysis using Cox proportional hazard models (Table 2), we observed increasing incidence trends for ESRD with impaired performance in the TUG test. Compared to individuals with a TUG performance of < 10 s, individuals with a higher TUG test result showed an increased risk of ESRD. In the fully adjusted model (Model 4), the adjusted HRs were 1.07 (95% CI 1.00–1.15) in the 10–14.9 s TUG test group, 1.11 (95% CI 0.96–1.30) in the 15–19.9 s TUG test group, and 1.45 (95% CI 1.08–1.95) in the > 20 s TUG test group. The overall increasing incidence trends for ESRD with impaired performance on the TUG test were maintained with the competing risk model.

**Table 2:** Incidence of ESRD with TUG categories

TUG	Subjects	ESRD incidence	Incidence rate^a^	Model 1	Model 2	Model 3	Model 4	Competing risk model
< 10	1,159,126	2732	0.47	1 (ref.)	1 (ref.)	1 (ref.)	1 (ref.)	1 (ref.)
≥10	393,655	1206	0.59	1.21 (1.13–1.33)	1.25 (1.16–1.33)	1.10 (1.02–1.17)	1.09 (1.12–1.17)	1.07 (1.00–1.15)
< 10	1,159,126	2732	0.47	1 (ref.)	1 (ref.)	1 (ref.)	1 (ref.)	1 (ref.)
10–14.9	340,276	984	0.55	1.15 (1.07–1.24)	1.18 (1.10–1.27)	1.07 (0.99–1.15)	1.07 (1.00–1.16)	1.07 (0.99–1.15)
15–19.9	45,684	177	0.71	1.46 (1.25–1.69)	1.51 (1.29–1.75)	1.19 (1.02–1.38)	1.11 (0.96–1.30)	1.08 (0.92–1.26)
≥20	7695	45	1.10	2.25 (1.68–3.02)	2.34 (1.74–3.14)	1.47 (1.10–1.98)	1.45 (1.08–1.95)	1.25 (0.91–1.73)

Model 1: crude model; Model 2: adjusting for sex, smoking status, alcohol history, exercise level, and income; Model 3: adjusting for variables of Model 2 with BMI, DM, HT, dyslipidemia, and eGFR; Model 4: adjusting for variables of Model 3 and state of dipstick proteinuria (4 categories); Competing risk model: adjusting for variables in Model 4 and considering competing risk

*BMI* body mass index, *DM* diabetes mellitus, *eGFR* estimated glomerular filtration rate using the abbreviated Modification of Diet in Renal Disease formula, *ESRD* end-stage renal disease, *HT* hypertension, *TUG* timed up-and-go test

^a^Per 1000 people

### Stratified and sensitivity analyses

Generally, increased incidence rate trends for ESRD with a poor TUG test performance were maintained in the stratified analysis according to varying baseline eGFR using the MDRD formula (Table 3). Moreover, these trends were maintained even in stratified analysis using eGFR based on CKD-EPI equations (Table 4).

**Table 3:** Stratified analysis of ESRD incidence according to baseline eGFR and TUG categories

eGFR (MDRD)	TUG	Subjects	ESRD incidence	Incidence ratio^a^	Model 1	Model 2	Model 3	Model 4	Competing risk model
15–29	−10	1713	659	104.26	1 (ref.)	1 (ref.)	1 (ref.)	1 (ref.)	1 (ref.)
	10–14.9	627	223	97.53	0.94 (0.81–1.09)	0.98 (0.84–1.14)	0.96 (0.82–1.11)	0.99 (0.85–1.15)	0.96 (0.82–1.12)
	151–9.9	112	52	124.65	1.20 (0.90–1.59)	1.32 (0.99–1.75)	1.26 (0.95–1.68)	1.19 (0.89–1.58)	1.12 (0.86–1.47)
	20–	33	11	105.54	1.02 (0.56–1.86)	1.32 (0.73–2.40)	1.12 (0.62–2.05)	1.41 (0.78–2.58)	0.76 (0.37–1.58)
30–59	−10	114,180	1120	1.89	1 (ref.)	1 (ref.)	1 (ref.)	1 (ref.)	1 (ref.)
	10–14.9	37,043	413	2.04	1.05 (0.93–1.17)	1.12 (1.00–1.25)	1.02 (0.91–1.14)	1.04 (0.93–1.16)	1.03 (0.92–1.16)
	15–19.9	5510	71	2.25	1.13 (0.89–1.44)	1.22 (0.96–1.56)	1.03 (0.81–1.32)	0.97 (0.76–1.23)	0.93 (0.72–1.21)
	20–	1053	16	2.72	1.37 (0.84–2.25)	1.55 (0.95–2.55)	1.08 (0.66–1.77)	1.00 (0.61–1.64)	0.94 (0.56–1.58)
60–89	−10	798,406	802	0.20	1 (ref.)	1 (ref.)	1 (ref.)	1 (ref.)	1 (ref.)
	10–14.9	219,894	284	0.25	1.19 (1.04–1.36)	1.21 (1.05–1.38)	1.16 (1.01–1.33)	1.13 (0.99–1.30)	1.11 (0.97–1.28)
	15–9.9	29,361	48	0.30	1.38 (1.03–1.85)	1.40 (1.04–1.87)	1.31 (0.97–1.75)	1.24 (0.93–1.66)	1.22 (0.90–1.64)
	20–	4851	15	0.57	2.67 (1.60–4.46)	2.71 (1.63–4.53)	2.41 (1.44–4.01)	2.15 (1.29–3.59)	2.09 (1.25–3.50)
≥90	−10	244,827	151	0.13	1 (ref.)	1 (ref.)	1 (ref.)	1 (ref.)	1 (ref.)
	10–14.9	82,712	64	0.15	1.18 (0.88–1.57)	1.20 (0.89–1.60)	1.17 (0.87–1.57)	1.16 (0.87–1.55)	1.15 (0.86–1.56)
	15–19.9	10,701	6	0.11	0.83 (0.37–1.88)	0.86 (0.38–1.94)	0.81 (0.36–1.82)	0.81 (0.36–1.83)	0.83 (0.37–1.87)
	20–	1758	3	0.34	2.59 (0.82–8.11)	2.66 (0.85–8.36)	2.53 (0.81–7.93)	2.52 (0.80–7.90)	2.48 (0.79–7.79)

Model 1: crude model; Model 2: Adjusting for sex, smoking status, alcohol history, exercise level, and income; Model 3: adjusting for variables of Model 2 with BMI, DM, HT, dyslipidemia, and eGFR; Model 4: adjusting for variables of Model 3 and state of dipstick proteinuria (4 categories); Competing risk model: adjusting for variables in Model 4 and considering competing risk

*BMI* body mass index, *DM* diabetes mellitus, *eGFR* estimated glomerular filtration rate using the abbreviated Modification of Diet in Renal Disease formula, *ESRD* end-stage renal disease, *HT* hypertension, *TUG* timed up-and-go test

^a^Per 1000 person-years

**Table 4:** ESRD incidence according to baseline eGFR and TUG categories, using the CKD-EPI eGFR formula

eGFR (CKD-EPI)	TUG	Subjects	ESRD incidence	Incidence ratio^a^	Model 1	Model 2	Model 3	Model 4
15–29	−10	1868	699	101.09	1 (ref.)	1 (ref.)	1 (ref.)	1 (ref.)
	10–14.9	671	239	97.19	0.96 (0.82–1.12)	1.00 (0.87–1.16)	0.98 (0.84–1.13)	1.2 (0.88–1.18)
	15–19.9	125	55	115.72	1.14 (0.87–1.50)	1.20 (0.91–1.58)	1.21 (0.92–1.60)	1.17 (0.88–1.54)
	20-	34	11	102.72	1.03 (0.57–1.87)	1.28 (0.70–2.33)	1.11 (0.61–2.02)	1.43 (0.78–2.60)
30–59	−10	114,006	1078	1.82	1 (ref.)	1 (ref.)	1 (ref.)	1 (ref.)
	10–14.9	36,997	397	1.96	1.04 (0.93–1.17)	1.11 (0.99–1.25)	1.02 (0.91–1.14)	1.02 (0.91–1.15)
	15–19.9	5496	68	2.16	1.12 (0.88–1.43)	1.21 (0.95–1.55)	1.07 (0.84–1.37)	0.97 (0.76–1.24)
	20-	1052	16	2.72	1.42 (0.87–2.33)	1.60 (0.97–2.61)	1.10 (0.67–1.80)	1.00 (0.61–1.64)
60–89	−10	643,658	710	0.22	1 (ref.)	1 (ref.)	1 (ref.)	1 (ref.)
	10–14.9	171,381	246	0.27	1.20 (1.04–1.39)	1.21 (1.04–1.40)	1.16 (1.00–1.34)	1.13 (0.98–1.31)
	15–19.9	22,505	43	0.34	1.45 (1.07–1.98)	1.46 (1.07–1.98)	1.36 (1.00–1.85)	1.29 (0.95–1.75)
	20-	3684	13	0.64	2.72 (1.57–4.71)	2.74 (1.58–4.74)	2.43 (1.41–4.22)	2.13 (1.23–3.70)
≥90	−10	399,594	245	0.13	1 (ref.)	1 (ref.)	1 (ref.)	1 (ref.)
	10–14.9	131,227	102	0.15	1.18 (0.94–1.49)	1.18 (0.94–1.49)	1.15 (0.91–1.44)	1.13 (0.90–1.43)
	15–19.9	17,558	11	0.12	0.92 (0.50–1.68)	0.92 (0.50–1.68)	0.87 (0.47–1.58)	0.86 (0.47–1.57)
	20-	2925	5	0.34	2.60 (1.07–6.30)	2.56 (1.06–6.21)	2.37 (0.98–5.74)	2.39 (0.99–5.80)

Model 1: crude model; Model 2: adjusting for sex, smoking status, alcohol history, exercise level, and income; Model 3: adjusting for variables of Model 2 with BMI, DM, HT, dyslipidemia, and eGFR; Model 4: adjusting for variables of Model 3 and state of dipstick proteinuria (4 categories)

*BMI* body mass index, *CKD-EPI* Chronic Kidney Disease Epidemiology Collaboration equation based on serum creatinine, *DM* diabetes mellitus, *eGFR* estimated glomerular filtration rate, *ESRD* end-stage renal disease, *HT* hypertension, *TUG* timed up-and-go test

^a^Per 1000 person-years

## Discussion

In this study, we found that baseline TUG performance was associated with a future incidence risk of ESRD in a dose-dependent manner among individuals in a large-scale 66-year-old Korean sample population. To our knowledge, this is the first study to show an association between physical frailty and the progression of CKD.

The association of impaired physical performance at baseline and the increasing incidence of ESRD might be explained through several pathophysiological mechanisms. ESRD is a well-recognized end phenotype of vascular aging pathology and inflammation that might be related to life-long cumulative exposure to metabolic stressors related to epigenetic (i.e. lifestyle) factors [6, 28, 29]. Cumulative evidence from basic research and clinical studies suggest that lifestyle-related chronic diseases are associated with both CKD progression and aging phenotypes, including frailty and cognitive dysfunction [6, 7, 30]. This inference is in accordance with that of a previous cross-sectional study using National Health and Nutrition Examination Survey (NHANES) subpopulation data that showed an association between lower physical activity levels and reduced kidney function [31]. Moreover, individuals in our study with a poor TUG test performance at examination had a higher burden of chronic metabolic diseases, and therefore, might have a higher degree of systemic aging than individuals with better TUG test performances, leading consequently to a higher incidence of ESRD during follow-up.

Although not evaluated in the present study, polypharmacy, a common geriatric syndrome defined as taking multiple medications, is frequently observed in older patients treated for multimorbidity. Polypharmacy might have contributed to worsening kidney function during the follow-up period [32]. The Atherosclerosis Risk in Communities cross-sectional study showed that frailty and polypharmacy were associated with reduced kidney function [33]. The association between polypharmacy and lower kidney function has been similarly observed in another cross-sectional study analyzing NHANES data [34]. In accordance with these study findings and, as physical frailty has been shown to overlap with multimorbidity and disabilities [9], impaired baseline physical performance determined as prolonged TUG result may imply a higher chronic disease burden and medication exposure of the kidneys.

Previous studies have shown associations between vascular aging phenotypes, such as small vessel disease and retinal vessel abnormalities, and physical performance [13, 35], which, in turn, support studies that have shown the effects of baseline frailty in patients with ESRD on clinical adverse outcomes [16, 17, 19], and associations between physical performance and the degree of CKD [36]. However, in our results, an impaired TUG performance increased the incidence of future ESRD, even in individuals with a GFR of 60–90 mL/min, suggesting life-long accumulation of subclinical interactions between the aging process and polypharmacy with multimorbidity and physical frailty.

Based on our findings, when managing older patients with CKD, it might be beneficial to incorporate physical performance and frailty status assessment in clinical practice, either for risk prediction or as a preventive measure for adverse outcomes. In addition to addressing frailty only as a risk factor, several studies have reported that physical frailty can be improved with structured physical activity programs or multicomponent intervention programs [37–39]. Through combining previous results concerning the relevance of frailty in patients with CKD in our study findings, a specifically designed multicomponent intervention with tailored care for patients with CKD and comorbidities may show improved clinical outcomes in this population.

In this study, the TUG test was used as a measure for physical performance to determine physical frailty. Despite the presence of the floor effect and difficulties in separating components of gait or balance from the overall score, the TUG test has been validated as a reliable measure of mobility and cognitive impairment [40]. The TUG test has been proposed as a measure of physical performance, which has been defined as objectively measured whole-body function related to locomotion in the recently updated European Working Guideline for Sarcopenia in Older People (EWGSOP2) [41]. Bischoff et al. [42] found that 92% of community-dwelling older women aged 65–85 years were able to perform the test in ≤12 s. In the EWGSOP2, TUG test of ≥20 s was suggested as a criterion for impaired physical performance. In our study, we categorized TUG test performance into to 4 groups, to evaluate the dose-response effect of TUG time on the incidence of ESRD.

This study was a nationwide, large, unselected population study of Korean older adults; however, it had some limitations. First, causality could not be determined in our observational study. While the associations were maintained, even after adjusting for eGFR as a continuous variable and the state of proteinuria in multivariable analysis, possibilities of reverse causality that impaired the TUG test results as consequences of increased CKD progression might have been reduced. Second, we used the eGFR based on serum Cr in this study, a biomarker that is processed in skeletal muscle, hence affected by skeletal muscle mass [43, 44]. Since muscle mass is correlated with muscle performance, and decreased muscle mass tends to result in a higher eGFR, it is possible that the association between decreased physical performance and a lower eGFR might have been diluted owing to the use of the eGFR value based on serum Cr. This may have in turn resulted in an incomplete adjustment of the baseline eGFR in our multivariable analysis. We used the CKD-EPI formula to assess the sensitivity of the MDRD formula; however, further assessments using a cystatin C-based eGFR, which we could not conduct using the current dataset, may resolve this weakness. Third, we relied on the TUG test results; however, we could not distinguish domains of physical performance, such as gait speed or grip strength to further assess component-specific relevance. Further studies using more detailed measures such as a short physical performance battery may reveal more information concerning this issue. Fourth, while we attempted to consider possible risk factors and diseases that might have affected renal function in our analyses, there might be unadjusted confounders that were not available in the NHIS database. Lastly, although the risk of ESRD was 1.45 times higher in the ≥20 s TUG test group compared to that in the < 10 s TUG test group, the absolute risk difference was notably low (< 1 per 1000 person-years) because of the low incidence rate of ESRD. While our data add evidence for the pathophysiological link between frailty and renal function, the clinical significance and utility of the TUG test in terms of renal care should be evaluated in further studies.

## Conclusions

In conclusion, using nationwide population-based examination data and prospective outcomes derived from claims data, we found that a poor TUG performance was associated with an increased risk in the incidence of ESRD, suggesting possible interactions between systemic frailty and vascular aging processes. Further research is warranted to determine whether a structured multicomponent intervention to prevent the progression of frailty in older patients with CKD can improve clinical outcomes.

## Data Availability

The datasets analysed during the current study are available in the Korean National Health Insurance Sharing Service (NHISS) repository, https://nhiss.nhis.or.kr/bd/ay/bdaya001iv.do.

## Abbreviations

BP: Blood pressure
CKD: Chronic kidney disease
CKD-EPI: Chronic kidney disease epidemiology collaboration
Cr: Creatinine
DM: Diabetes mellitus
ESRD: End-stage renal disease
eGFR: Estimated glomerular filtration rate
HRs: Hazard ratios
HDL: High-density lipoprotein
HT: Hypertension
ICD-10: International classification of diseases 10th revision
IRB: Institutional review board
KNHIS: Korean national health insurance service
LDL: low-density lipoprotein
NHANES: National health and nutrition examination survey
NHSP: National health screening program
NSPTA: National screening program for transitional ages
MDRD: Modification of diet in renal disease
TUG: Timed up-and-go
